# Prospective observational cohort of algorithm-guided reassessment in diabetic foot: real-world outcomes and prognostic associations

**DOI:** 10.3389/fcdhc.2026.1799455

**Published:** 2026-04-15

**Authors:** João Antônio Correa, Gabriela Tessaro Cremoneis, Paula Stefany Santos Caetano, Isabela Yones Nogara, Sidnei José Galego, Rafael Vilhena de Carvalho Furst, João Paulo Tardivo, Rodrigo Daminello Raimundo

**Affiliations:** 1 Department of Vascular Surgery, Centro Universitário FMABC, Santo André, SP, Brazil; 2 Laboratory of Research Design and Scientific Writing, Centro Universitário FMABC, Santo André, SP, Brazil

**Keywords:** cohort, diabetic foot, lower-limb amputations, peripheral arterial disease, Tardivo algorithm

## Abstract

**Background:**

Diabetic foot is among the most frequent complications of diabetes mellitus (DM), with potentially dramatic consequences ranging from chronic wounds to major lower-limb amputations. The Tardivo Algorithm is a simple prognostic scoring system designed to support risk stratification and structured longitudinal reassessment in routine clinical care.

**Objective:**

To describe the real-world implementation and feasibility of dynamic risk reassessment using the Tardivo Algorithm in a prospective observational cohort of patients with diabetic foot managed in a vascular surgery outpatient setting, and to explore associations between baseline risk stratification and clinical outcomes.

**Methods:**

This prospective observational cohort study was conducted in a routine outpatient clinic for complex wounds. Adult patients with diabetic foot were classified according to the Tardivo Algorithm at baseline and underwent structured serial reassessments at each follow-up visit as part of usual multidisciplinary care. No comparator group was included. Patients were followed for 6–18 months, and outcomes were descriptively recorded as minor amputation, major amputation, wound in process of healing, or complete healing.

**Results:**

A total of 42 patients were followed for up to 18 months. Mean initial Tardivo score was 7.6 ± 4.8, with 19% classified as high risk (≥12 points). Limb preservation was observed in 94.3% of participants, and complete healing occurred in 57%, with a mean healing time of 5.05 ± 1.95 months. Higher baseline Tardivo scores were positively associated with peripheral arterial disease (r = 0.740; p < 0.001), while healing time correlated with both PAD (r = 0.547; p = 0.006) and prior amputations (r = 0.523; p = 0.009). These correlations were not independent in multivariable models. Findings reflect associations observed in a real-world, structured outpatient care model.

**Conclusion:**

In this prospective real-world cohort, structured application of the Tardivo Algorithm was feasible and allowed dynamic clinical monitoring. Clinical outcomes observed during follow-up are described within the context of this non-controlled design and should be interpreted as observational associations rather than indicators of therapeutic effect. Controlled studies are required to determine the independent impact of algorithm-guided reassessment and adjunctive therapies.

## Introduction

Diabetes mellitus (DM) is a chronic, progressive disease affecting millions worldwide. It is associated with microvascular complications—such as nephropathy, retinopathy, and diabetic neuropathy—and macrovascular complications, including coronary artery disease and peripheral vascular disease. These complications signal an advanced stage of illness and substantially increase the risk of adverse outcomes, including development of diabetic foot, one of the leading causes of non-traumatic lower-limb amputation (1).

Diabetic foot is defined as infection, ulceration, and/or destruction of deep tissues of the foot, frequently associated with peripheral neuropathy and varying degrees of peripheral vascular disease (2). This condition is one of the most common and disabling complications of diabetes, with major impact on patients’ quality of life. An estimated 50–60% of diabetic foot ulcers become infected, and about 20% of moderate-to-severe infections progress to amputation. Five-year mortality is approximately 30% among patients with ulcers and exceeds 70% among those undergoing major amputation (3).

Given the rising prevalence of diabetes and its complications, several therapeutic strategies have been developed to optimize treatment, reduce amputation rates, and improve ulcer healing. One such tool is the Tardivo Algorithm, published in 2015 by João Paulo Tardivo and colleagues. This assessment model aims to predict amputation risk in individuals with diabetic foot by considering three main parameters (1): the Meggitt–Wagner classification (2); signs of peripheral arterial disease; and (3) ulcer location on the foot. The final score ranges from 1 to 32, with higher values indicating worse prognosis. Scores of 2–7 indicate low risk, 8–11 intermediate risk, and ≥12 high risk for amputation (4).

A multidisciplinary approach is essential for effective management of diabetic foot. Engagement of specialists—such as infectious disease physicians, vascular surgeons, podiatrists, and primary care providers—has been associated with significant reductions in amputation rates (1, 3). Approximately 30–40% of ulcers heal within 12 weeks; however, recurrence is high, reaching 42% in the first year and up to 65% within five years (3).

Standard treatment for diabetic foot includes therapeutic footwear, specialized dressings, antibiotics, debridement, and revascularization when indicated. Novel therapies under investigation include acellular dermal matrices, topical oxygen, ultrasonic debridement, negative-pressure wound therapy, and hyperbaric oxygen therapy (3).

Among innovative options, photodynamic therapy (PDT) is a relatively underexplored method for diabetic foot management. PDT uses a photosensitizing agent activated by a specific light source to modulate immune responses and induce cell death, aiding in control of local infection. It is low-cost, safe, and applicable to ischemic and non-ischemic patients (5).

Another promising alternative is the use of platelet-rich fibrin (PRF) membranes, a matrix containing platelet-derived growth factors that promote cell adhesion and proliferation. PRF has been widely used for tissue regeneration and has shown positive results in chronic, hard-to-heal ulcers, including vascular ulcers and diabetic foot ulcers. Developed by Choukroun et al. in 2000, PRF is easy to obtain, low-cost, and requires no chemical additives. We currently employ the PRO-PRF technique, developed by Saboia-Dantas and Dechichi, which yields a firmer membrane that facilitates application to wounds (6–8).

In this context, structured and reproducible tools may support longitudinal risk reassessment and standardized clinical monitoring in diabetic foot management. This study aimed to describe the real-world implementation of the Tardivo Algorithm in a prospective cohort and to explore its association with clinical evolution under routine multidisciplinary care.

## Methods

This prospective observational cohort study was conducted in a routine outpatient clinic for complex wounds. Adult patients with diabetic foot were classified according to the Tardivo Algorithm at baseline and underwent structured serial reassessments at each follow-up visit as part of usual multidisciplinary care. No comparator group was included. Patients were followed for 6–18 months, and outcomes were descriptively recorded as minor amputation, major amputation, wound in process of healing, or complete healing. The study was conducted at the Centro Hospitalar Municipal de Santo André, in the complex-wound outpatient clinic for diabetic foot managed by the Vascular Surgery team, in Santo André, São Paulo State, Brazil, affiliated with the FMABC School of Medicine and the Fundação do ABC. The study was approved by the Research Ethics Committee (#6.130.334; CAAE: 68109722.1.0000.0082).

### Participants

The Centro Hospitalar Municipal de Santo André has a Vascular Surgery team specializing in diabetic foot care. Patients from this clinic who met inclusion criteria did not meet exclusion criteria, and agreed to participate were enrolled. A total of 42 patients participated. All procedures were performed in the outpatient clinic. Data collection occurred from November 2022 to May 2024 (total study period of 16 months). Individual follow-up duration varied according to time of enrollment and clinical evolution. Participants were adults (≥18 years) of either sex with diabetic foot and a trophic lesion under outpatient follow-up who were receiving photodynamic therapy (PDT) and/or platelet-rich fibrin (PRF) and provided informed consent. Patients were excluded if they had Wagner grade 0 or 5, were hospitalized at screening, or had lesions due to etiologies other than diabetic foot.

For limb-preservation analysis (event = major amputation), patients were censored at the date of last outpatient contact if they were lost to follow-up, died from causes unrelated to diabetic foot, or reached the end of the observation period without major amputation. For time-to-healing analyses (event = complete healing), individuals who did not achieve complete healing were censored at their last contact date; major amputation and death were treated as censoring events (or, alternatively, as competing events—see limitation).

### Assessment and interventions

At the first visit, patients underwent interviews, history, physical examination, and were classified using the Tardivo Algorithm. Lesions were assessed at the procedure tables; dressings were removed and wound care performed: cleansing with antiseptic and saline, devitalized tissue, removal of hyperkeratosis and biofilm. The appropriate therapy—simple dressing, PDT, or regenerative therapy with PRF membrane—was then selected.

When indicated, procedures were performed and the wound was covered with a simple dressing. Family members and patients received care instructions; antibiotics, symptom control, and special footwear were prescribed as needed. For study integrity, patients were expected to attend scheduled visits to allow evaluation and to follow team recommendations.

Adjunctive therapies (PDT and/or PRF) were applied according to clinical judgment, wound characteristics, and resource availability. No predefined protocol determined allocation between PDT alone, PRF, or combined therapy. In general, PDT was preferentially used in wounds with signs of infection or biofilm, whereas PRF was considered for chronic non-healing wounds after infection control. The Tardivo score functioned primarily as a prognostic and monitoring tool, and treatment decisions were based on comprehensive clinical assessment rather than exclusively on risk category.

### Tardivo algorithm

Published in 2015 by J. P. Tardivo et al. (4), the Tardivo Algorithm is a simple assessment system designed to predict amputation risk and facilitate management of diabetic foot. Developed from a sample of 62 patients, it is based on three parameters (1): Meggitt–Wagner grade (2); signs of peripheral arterial disease (PAD); and (3) ulcer location. The final score is the product of scores in each domain and ranges from 1 to 32, with higher scores indicating worse prognosis. Scores 2–7 indicate low risk, 8–11 intermediate risk, and ≥12 high risk of amputation.

The Meggitt–Wagner grade is a linear scale from 0 to 5 based primarily on depth, and only grades 1–4 are considered in the algorithm, receiving coincident scores from 1 to 4. Peripheral arterial disease (PAD) classification relies on the presence of ischemic signs—such as pallor, non-palpable distal pulses, ankle–brachial index <0.7, absent digital perfusion, fixed cyanosis, or dry gangrene—so good peripheral perfusion without ischemic signs is scored as 1 (PAD = 1), whereas any ischemic sign is scored as 2 (PAD = 2). Ulcer location is categorized by dividing the foot into four regions—forefoot 1 (phalanges), forefoot 2 (metatarsals), midfoot 3 (cuneiforms, cuboid, naviculars), and hindfoot 4 (calcaneus and talus)—and the region number is used as the score for this domain.

The Tardivo score was used as a prognostic and monitoring tool. Therapeutic decisions were primarily based on clinical evaluation rather than solely on risk category.

Adjunctive therapies [photodynamic therapy (PDT) and/or platelet-rich fibrin (PRF)] were applied based on clinical judgment within the multidisciplinary team. Allocation was not determined by a predefined protocol or by Tardivo risk category alone. Decisions considered wound characteristics, including signs of infection, tissue viability, exudate, depth, and response to prior treatment. In general, PDT was preferentially used in wounds with suspected local infection or high bioburden, whereas PRF was applied in clean, granulating wounds to support tissue regeneration. When both therapies were used, PRF was typically applied after local infection control had been achieved with PDT.

Worsening of the Tardivo score was defined as any increase in the absolute score compared with baseline during follow-up. Because the score is derived from Wagner grade, PAD status, and ulcer location, an increase reflected clinical progression in at least one component. Worsening was defined by numerical score increase rather than solely by change in categorical risk classification.

### Photodynamic therapy

PDT combines a photosensitizer with specific light to generate reactive oxygen species with potent cytotoxic effects against bacteria, fungi, protozoa, and viruses. It can be applied in chronic infections and hard-to-heal wounds, such as diabetic foot ulcers (5, 8, 9). In this study, methylene blue solution served as the photosensitizer (two 5-mL ampoules of 2% methylene blue diluted in 250 mL saline), applied to wound beds with syringes and into cavities/fistulous tracts using probes. Before irrigation with the solution, hydrogen peroxide was applied to facilitate agent distribution. A red LED plate (50 mW; 30 J/cm² for 10 minutes) was then placed in contact with the wound, followed by a simple dressing.

### Platelet-rich fibrin regenerative matrix

PRF membranes leverage platelet-derived growth factors to accelerate healing of chronic wounds, including diabetic ulcers (6). We used the third-generation membrane technique (Saboia-Dantas and Dechichi). Venous blood was drawn and distributed into additive-free tubes for centrifugation over 15 minutes with stepwise speed increases (700/1300/2400 RPM). The supernatant was removed and dehydrated to a gelatinous consistency for application to the wound bed. Liquid PRF was injected into wound edges; the membrane was fixed to the margins with cyanoacrylate, then covered with PVC and a bandage. The dressing remained for one week; the secondary layer could be changed as needed, and patients returned for removal and reassessment (6, 7).

### Statistical analysis

Data were organized in Microsoft Excel and analyzed using SPSS version 22.0 (Chicago, IL, USA). Continuous variables were tested for normality using the Shapiro–Wilk test and are presented as mean ± standard deviation when normally distributed. Categorical variables are presented as absolute and relative frequencies. Continuous variables included age, duration of diabetes, baseline Tardivo score, and time to complete healing (in months). Binary variables (coded as 0/1) included peripheral arterial disease (PAD), prior minor amputation, PRF use, and PDT use. Bivariate associations between continuous variables were assessed using Pearson’s correlation coefficient. For associations between continuous and binary variables, Pearson correlation was used, which is mathematically equivalent to the point-biserial correlation coefficient when binary variables are coded as 0/1. Ninety-five percent confidence intervals (95% CI) for correlation coefficients were calculated using Fisher’s z transformation. All analyses were conducted using pairwise complete cases. Proportions (e.g., complete healing, major amputation, limb preservation) are reported with exact 95% confidence intervals. To evaluate independent predictors of complete healing, a multivariable logistic regression model was constructed including age, duration of diabetes, PAD, prior minor amputation, and baseline Tardivo score. These variables were selected *a priori* based on clinical relevance. Adjusted odds ratios (OR) with 95% CIs are reported. Among patients who achieved complete healing, a multivariable linear regression analysis was performed to explore predictors of time to healing, using the same covariates. Regression coefficients (β) with 95% CIs are reported. Given the low number of major amputation events (n=2) and incomplete time-to-event data, survival analysis and multivariable modelling for this outcome were not performed to avoid unstable estimates and risk of overfitting. All p-values are two-sided, and values <0.05 were considered statistically significant.

## Results

The sample was predominantly male (80.9%), with a mean age of 60 ± 9.9 years (range 39–83). Mean duration of diabetes was 16.5 ± 8.5 years. Overall, 69% had at least one comorbidity, most commonly systemic arterial hypertension (57%). Peripheral arterial disease (PAD) was present in 21.4% of participants, and 40.4% had a history of prior minor amputation.

The mean baseline Tardivo score was 7.6 ± 4.8. Eight patients (19%) were classified as high risk (≥12 points). Complete healing occurred in 24 of 42 patients (57.1%; 95% CI 40.9%–72.3%). Major amputation occurred in 2 patients (4.8%; 95% CI 0.6%–16.2%), and limb preservation was achieved in 40 of 42 individuals (95.2%; 95% CI 83.8%–99.4%) during the observation period. Among high-risk patients (n=8), 7 avoided major amputation during follow-up (87.5%; 95% CI 47.3%–99.7%). Given the small subgroup size, this finding should be interpreted descriptively.

In unadjusted analyses, higher initial Tardivo score correlated positively with PAD (r=0.740; p<0.001) and prior amputation (r=0.523; p=0.009). Healing time correlated positively with PAD (r=0.547; p=0.006) and prior amputation (r=0.523; p=0.009). Other comorbidities observed included obesity, chronic kidney disease, heart failure, and hypothyroidism (**Table 1**).

**Table 1:** Demographic and epidemiological profile of patients.

Variable	Male (Mean ± SD)	Female (Mean ± SD)	Total
Age (years)	59 ± 8.36	63.8 ± 15.3	60 ± 9.9
Average time of diabetes mellitus diagnosis (years)	15.4 ± 11.1	22 ± 8.5	16.5 ± 8.51
	N (%)	N (%)	N (%)
Insulin-dependent diabetes	19 (45.2)	5 (11.9)	24 (57.1)
Associated comorbidities	22 (52,3)	7 (16.6)	29 (69)
Previous amputations	17 (40.4)	0	17 (40.4)
PAD presence	9 (21.4)	0	9 (21.4)
Total	34 (80.9)	8 (19.1)	42 (100)

N, number of patients; %, percentage; SD, standard deviation; PAD, peripheral arterial disease.

Only one patient had undergone revascularization prior to study inclusion. The most common baseline Tardivo score was 6 (38%). Risk stratification classified 25 patients as low risk, 9 as intermediate, and 8 as high risk.

Among the 42 participants, 37 (88%) received PDT (mean 4.54 sessions); 28 (66%) underwent ≤5 PDT sessions. Eleven (26%) received PRF; 8 received one session, 1 received two, and 2 received three.

Worsening of the Tardivo score during follow-up was observed in 9 individuals (21%). Of these, 2 (4%) underwent major amputation. Four achieved complete healing (mean 6.25 months), and three remained under follow-up.

Among high-risk patients (initial score ≥12), 4 of 7 (57%) showed score increases during follow-up; 5 (71%) achieved complete healing; 1 required major amputation; and 1 remained under follow-up. All were male (mean age 63.8 years).

Mean time to complete healing among those who healed (n=24) was 5.05 ± 1.95 months (range 2–10). Among these, women had a mean healing time of 3.25 months and men 4.95 months.

At the end of 16 months of data collection, 2 patients (4.7%) had undergone major amputation, 1 (2.3%) died from unrelated causes, and 6 (14%) were lost to follow-up. Of the remaining, 9 had not achieved complete healing, but most showed favorable evolution with a mean follow-up of 10.2 months.

### Multivariable analysis

In logistic regression adjusting for age, diabetes duration, PAD, prior minor amputation, and initial Tardivo score, no independent predictor of complete healing was identified. The adjusted OR for Tardivo score was 0.88 per point increase (95% CI: 0.70–1.11; p = 0.287). PAD had an elevated but imprecise association (OR 6.51; 95% CI: 0.44–96.59; p = 0.173) (**Table 2**).

**Table 2:** Multivariable logistic regression analysis of factors associated with complete healing.

Variable	Adjusted OR	95% CI	p-value
Age	1.00	0.93 – 1.08	0.952
DM duration	0.97	0.91 – 1.04	0.422
PAD	6.51	0.44 – 96.59	0.173
Prior minor amputation	0.68	0.17 – 2.67	0.580
Initial Tardivo score (per point)	0.88	0.70 – 1.11	0.287

DM, Diabetes mellitus; PAD peripheral arterial disease.

In patients who achieved complete healing, multivariable linear regression revealed no independent predictors of healing time. Prior amputation showed a borderline association with longer healing time (β = 1.29 months; 95% CI −0.15 to 2.72; p = 0.075). Tardivo score was not independently associated with healing time (β = 0.16; 95% CI −0.07 to 0.39; p = 0.155).

Due to the small number of major amputations (n=2), no regression was performed for that outcome.

### Discrimination analysis

Receiver operating characteristic (ROC) analysis showed an AUC of 0.57 for complete healing, indicating limited discriminative performance of the Tardivo score in this sample. For major amputation, AUC was 0.66; however, due to only two events, this estimate lacks reliability.

**Table 3** presents selected clinically relevant bivariate associations with corresponding 95% confidence intervals. There was a positive correlation between baseline Tardivo score and PAD (r=0.740; 95% CI 0.562–0.852; p<0.001), but not with prior minor amputation (r=0.140; 95% CI −0.171–0.426; p=0.377). Among patients who achieved complete healing, healing time correlated positively with PAD (r=0.547; 95% CI 0.184–0.779; p=0.006) and with prior minor amputation (r=0.523; 95% CI 0.151–0.765; p=0.009). Sex was examined as a potential covariate in the multivariable models. Its inclusion did not materially modify regression coefficients or improve model fit, and it was therefore not retained in the final adjusted analyses. .

**Table 3:** Correlation between the study variables.

Association	n	r	95% CI	p-value
Baseline Tardivo score vs PAD	42	0.740	0.562 to 0.852	<0.001
Baseline Tardivo score vs prior minor amputation	42	0.140	−0.171 to 0.426	0.377
Time to complete healing (months) vs PAD	24	0.547	0.184 to 0.779	0.006
Time to complete healing (months) vs prior minor amputation	24	0.523	0.151 to 0.765	0.009

Pearson correlation coefficients (equivalent to point-biserial correlation for binary variables coded as 0/1) are presented with two-tailed p-values and 95% confidence intervals calculated using Fisher’s z transformation. Analyses were performed using pairwise complete cases. PAD, peripheral arterial disease; PRF, Platelet Rich Fibrin; PDT, Photodynamic Therapy; PAD, peripheral arterial disease.

A *post hoc* power estimation indicated that the study had approximately 80% power to detect correlation coefficients ≥0.42 at α=0.05 (two-tailed).

**Figure 1:**
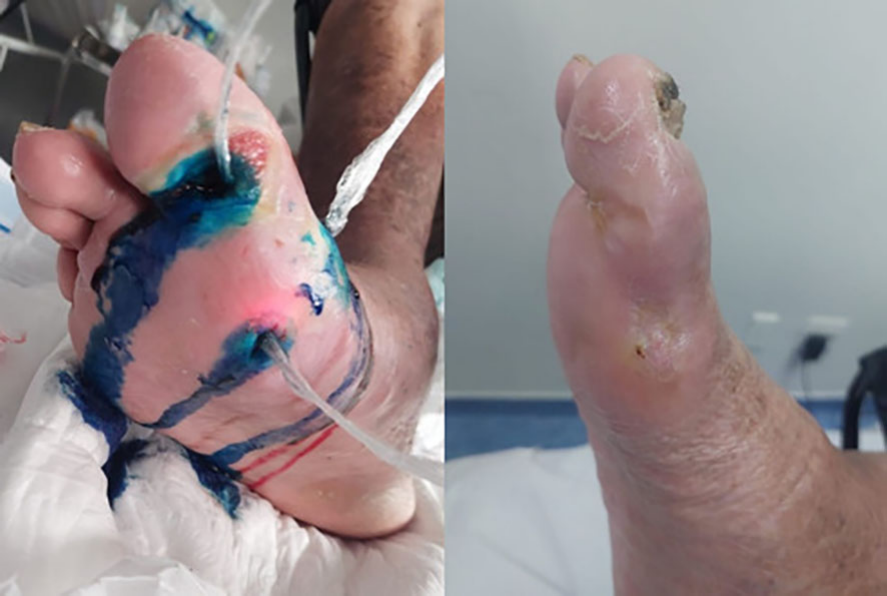
Hallux fistula associated with osteomyelitis—complete healing after photodynamic therapy. **Figure 1** illustrates the treatment course of a patient with diabetic foot and multiple fistulas involving the hallux and first metatarsal region, managed with fiber-assisted photodynamic therapy, resulting in complete wound healing and closure of the fistulas.

**Figure 2:**
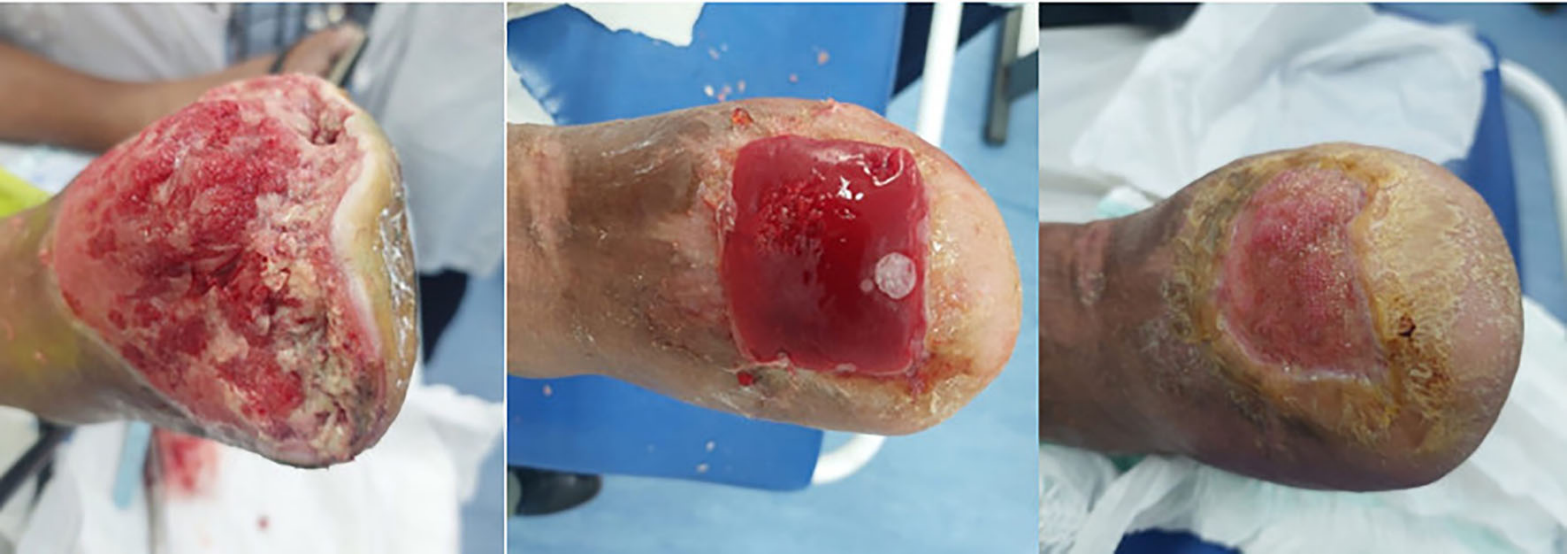
Evolution of a transmetatarsal amputation stump with an extensive lesion after photodynamic therapy, surgical debridement, and application of a platelet-rich fibrin membrane. **Figure 2** depicts the treatment course of a patient who underwent transmetatarsal amputation followed by multiple surgical debridements and photodynamic therapy. Regenerative therapy with a PRF membrane was then applied, leading to marked wound contraction and progressive superficialization of the lesion.

**Figures 1**, **2** illustrate representative cases of wound evolution under structured follow-up. These images are provided for illustrative purposes and do not constitute quantitative outcome measures.

## Discussion

This study describes the implementation of a structured, algorithm-guided reassessment model in a real-world outpatient cohort of patients with diabetic foot. Rather than testing therapeutic efficacy, the present findings illustrate the feasibility of dynamic risk stratification and its association with clinical outcomes over time. The observed associations between baseline Tardivo score, peripheral arterial disease, and healing time are consistent with the structural components of the scoring system and illustrate how the algorithm functioned within a structured outpatient monitoring model. These findings should not be interpreted as external validation of predictive performance but rather as observational coherence within routine clinical implementation.

Diabetes is one of the most prevalent chronic diseases today, intrinsically linked to obesity and sedentary lifestyle—trends increasingly common in modern society (10, 11). According to the World Health Organization, diabetic foot is characterized by ulceration, infection, and/or gangrene of the foot associated with diabetic neuropathy and varying degrees of peripheral arterial disease (12).

Among diabetes-related complications, diabetic foot is one of the most severe, often leading to hospitalization due to foot lesions that can serve as portals of entry for systemic infection and represent a major cause of major amputation. In addition, diabetic foot impairs quality of life due to reduced mobility and frequent foot care needs (daily dressing changes and continuous follow-up with vascular surgeons and wound-care specialists), increasing demands on health systems and family caregivers (10, 13). Strategies that avoid negative outcomes—such as major amputation and prolonged time to healing—can improve quality of life and reduce costs associated with extended care, potential hospitalizations, and absence from work by patients and caregivers (13–15).

The mean individual follow-up duration was 5.57 months within the 16-month study period. Initially, visits were weekly; intervals were lengthened based on clinical progress and shortened if deterioration occurred. This approach enabled close surveillance and rapid response to complications. Multidisciplinary care and close follow-up characterized the management model applied in this cohort.

At each visit, patients were reclassified using the Tardivo Algorithm, and potential benefit from PDT and/or PRF was reassessed. PDT was used in 37 patients at least once, and 66% received up to five sessions. In this cohort, 11 patients (26%) received both PDT and PRF, which may have synergistic effects—evidenced by reductions in ulcer size and exudate and increased granulation tissue. In chronic, hard-to-heal ulcers, PDT can decontaminate and condition the wound bed, followed by PRF to stimulate tissue regeneration.

In some cases, despite baseline scores <12 and absence of PAD, other factors likely contributed to prolonged healing: lesion-intrinsic characteristics, sites subjected to continuous mechanical stress, chronic infection and osteomyelitis, individual variability in treatment response, and comorbidities not captured by the score. Thus, even lower-risk patients may experience delayed healing, highlighting the complexity of clinical management and tissue repair.

Patients with diabetes should be evaluated by vascular surgeons at least annually, with preventive measures—inspection of the feet, patient/family education, appropriate footwear, neuropathy assessment, and risk-factor modification—particularly for the contralateral limb (1, 11, 16). Our results illustrate how structured, systematic, multidisciplinary follow-up can be operationalized in routine practice. Limb preservation (94.3%) and complete healing (57%) were observed during follow-up in this cohort. These figures are reported descriptively and should be interpreted within the methodological limits of a non-comparative cohort. Although these figures appear favorable when descriptively compared with published data, the absence of a comparator group precludes conclusions regarding therapeutic superiority. Rather, these findings illustrate the feasibility of structured algorithm-guided reassessment combined with individualized adjunctive therapies in a real-world outpatient setting.

The observed major amputation rate (4.7%) is described within the context of this single-center cohort. Given the observational design, it is not possible to isolate the independent contribution of algorithm-guided reassessment, adjunctive therapies, or multidisciplinary follow-up to this outcome. These findings suggest that early recognition of clinical deterioration within a structured follow-up framework may facilitate structured clinical decision-making within routine practice. However, given the observational design, it is not possible to isolate the independent contribution of PDT, PRF, or the algorithm itself to clinical outcomes.

This study was conducted in a single-center specialized vascular outpatient clinic with multidisciplinary expertise and access to adjunctive regenerative therapies. Therefore, direct extrapolation of clinical outcomes to primary care or resource-limited settings should be undertaken cautiously. While the Tardivo Algorithm relies primarily on clinical variables that may be applicable in broader healthcare contexts, implementation fidelity and therapeutic availability may differ substantially across systems. Future studies should evaluate structured algorithm-guided reassessment in diverse clinical environments to determine external validity.

Risk stratification systems for the diabetic foot have been studied in various cohorts, yet validation of predictive accuracy remains limited. A systematic review by Monteiro‑Soares et al. (17) showed that although several classification systems are widely used, few studies report formal diagnostic accuracy or external validation of prognostic performance. Prospective multicenter cohort studies have indicated that stratification tools can classify patients into different risk categories, but their performance may vary according to clinical setting and care level (18). Furthermore, systematic analyses of prognostic models for DFU progression to amputation reveal a wide range of model performance and emphasize methodological limitations that future research should address (19).

Our results may be better understood within the evolving literature on structured algorithms for diabetic foot care. Classical clinical algorithms have consistently emphasized that diabetic foot management should not rely solely on wound appearance, but rather on integrated assessment of perfusion, infection, depth, and anatomical location. In particular, Dovell and Hinchliffe (20) highlighted that vascular evaluation is a core step in diabetic foot pathways, because identification of peripheral arterial disease directly affects prognosis, urgency, and the need for revascularization. Similarly, Hong and Oh (21) described limb-salvage algorithms that combine wound severity and vascular status to guide staged decision-making, underscoring that standardized approaches may help clinicians recognize deterioration earlier and rationalize referral and treatment priorities. This perspective is consistent with our observation that baseline Tardivo score was strongly associated with PAD, one of the central determinants of worse evolution in diabetic foot.

At the same time, newer data suggest that algorithmic support in diabetic foot care is expanding beyond traditional rule-based models. Nanda et al. (22) reported that machine-learning methods may assist in identifying risk factors associated with diabetic foot ulcer severity, while the systematic review by Tulloch et al. (23) concluded that artificial intelligence and machine-learning tools show promise in prevention, diagnosis, and management, although methodological heterogeneity and limited external validation remain important barriers. In addition, Alsararatee et al. (24) recently reinforced the importance of structured diabetic foot assessment in inpatients, where delayed recognition of severity may worsen outcomes. Taken together, these studies support the broader principle that standardized and reproducible assessment frameworks are valuable in diabetic foot care (25–29). Our study adds to this discussion by showing that, even in a real-world outpatient cohort, repeated application of a simple bedside algorithm was operationally feasible and clinically coherent, although the present design does not permit conclusions regarding comparative predictive accuracy or therapeutic efficacy.

The most significant methodological limitation of this study is the absence of a control or comparator group. Without a parallel cohort receiving standard care without algorithm-guided reassessment, it is not possible to determine whether the observed outcomes were attributable to the Tardivo Algorithm, to adjunctive therapies (PDT and/or PRF), to multidisciplinary care, or simply to natural disease progression. Accordingly, the results should be interpreted as descriptive associations within a prospective cohort, rather than as evidence of therapeutic efficacy.

Adjunctive therapies were not standardized, and treatment allocation was based on clinical judgment. This introduces potential indication bias, as patients with more severe or refractory lesions may have been more likely to receive combined therapies. Consequently, differences in outcomes cannot be confidently attributed to specific interventions.

Due to the low number of major amputation events and incomplete time-to-event data for all participants, survival modeling was not performed, in order to avoid unstable estimates and overfitting. Future studies with larger samples and standardized follow-up intervals would enable more robust time-to-event analyses.

An additional limitation is the absence of standardized, quantitative wound area measurements. Although time to complete healing was recorded, objective methods such as digital planimetry were not employed prospectively. As a result, wound evolution is described primarily through clinical outcomes and illustrative images. Future research should incorporate standardized wound area assessments to more precisely evaluate tissue repair dynamics.

Given the observational nature of this study and the absence of a control group, future research should aim to build upon these preliminary findings through more rigorous designs. Randomized controlled trials are needed to evaluate the isolated efficacy of the Tardivo Algorithm and its associated adjunctive therapies (PDT and PRF). Additionally, external validation of the Tardivo score in different clinical environments—such as primary care settings, rural areas, or resource-limited outpatient services—would help determine its generalizability and operational utility beyond specialized vascular clinics. Incorporating standardized wound measurements and quality-of-life outcomes would further enhance the understanding of clinical benefit in future investigations.

## Conclusion

In this prospective real-world cohort, structured application of the Tardivo Algorithm was feasible and allowed dynamic clinical monitoring. Favorable outcomes were observed; however, these results represent observational associations within a non-controlled design. Controlled trials are necessary to determine the independent effect of algorithm-guided reassessment and adjunctive therapies.

## Data Availability

The raw data supporting the conclusions of this article will be made available by the authors, without undue reservation.
